# The human phageome: niche-specific distribution of bacteriophages and their clinical implications

**DOI:** 10.1128/aem.01788-24

**Published:** 2025-04-16

**Authors:** Izabela Rybicka, Zuzanna Kaźmierczak

**Affiliations:** 1 Laboratory of Phage Molecular Biology, Hirszfeld Institute of Immunology and Experimental Therapy 89217 , Wrocław, Poland; 2 Research and Development Center, Regional Specialist Hospital in Wrocław 49657 , Wrocław, Poland; 3 Faculty of Medicine, Department of Preclinical Sciences, Pharmacology and Medical Diagnostics, Wrocław University of Science and Technology 49567, Wrocław, Poland; Universidad de los Andes, Bogotá, Colombia

**Keywords:** bacteriophages, human microbiome, phageome, high-throughput sequencing, metagenomics

## Abstract

Bacteriophages (phages) play a crucial role in shaping the composition and diversity of the human microbiome across various body niches. Recent advancements in high-throughput sequencing technologies have enabled comprehensive analysis of the human phageome in different body sites. This review comprehensively analyzes phage populations across major human body niches, examining their distribution and dynamics through recent metagenomic discoveries. We explore how phage–bacteria interactions within different body sites contribute to homeostasis and disease development. Emerging evidence demonstrates that phageome perturbations can serve as early indicators of various disorders, particularly in the gut microbiome. Understanding these complex microbial interactions offers promising opportunities for developing novel diagnostic markers and therapeutic approaches. However, the causal relationship between phages, bacteria, and disease development remains unclear. Further research is needed to elucidate the role of phages in human health and disease and to explore their potential as diagnostic or therapeutic tools. Understanding the intricate interactions between phages, bacteria, and the human host is crucial for unraveling the complexities of the human microbiome and its impact on health and disease.

## INTRODUCTION

In recent years, there has been a tremendous increase in interest in human microbiome studies. The great part of this study area is covered by bacteria; however, bacteriophages (phages, viruses able to infect bacteria) also play a crucial role in this structure. Phages, as a significant part of the microbiome, have become the subject of increasing interest. Nevertheless, studies of the phageome remain challenging due to the great diversity of these viruses and lack of universal markers (unlike bacteria which have 16S rRNA for identification) (1). Traditional methods of phage identification based on bacterial host cultures have limited applicability for phage detection, mostly due to the significant contribution of laboratory unculturable or difficult-to-culture bacteria that are hosts for many phages constituting the phageome. These barriers have been overcome to some extent by the use of high-throughput DNA sequencing like next-generation sequencing (NGS). Application of this technology was a milestone in phageome studies (2). NGS has become the major tool for exploring and investigating phage presence in biological samples. Sequencing-based molecular methods have revealed that many human niches are inhabited by unique and place-specific microbiomes including phages (3). Due to the development of metagenomic profiling, comprehensive analysis of phages inhabiting different compartments of human bodies has become possible. These analyses demonstrated that phages are most abundant in the oral cavity, gastrointestinal tract, urinary tract, skin, and lungs (4).

This review examines phage populations across five major human body niches: gut, oral cavity, skin, respiratory tract, and urogenital tract. These environments harbor distinct microbial ecosystems, where bacteria and their associated phages engage in complex interactions that influence both microbial community dynamics and host health.

A significant challenge in phageome research lies in the evolving nature of viral taxonomy. Traditional classification systems based on morphology have proven inadequate for capturing the true complexity of phage relationships. This became particularly evident in *Caudovirales* taxonomy, where phages sharing similar genomic and functional characteristics were sometimes classified into different families based solely on morphological features (2, 5–7). Addressing these limitations, the International Committee on Taxonomy of Viruses (ICTV) introduced in 2020 a comprehensive 15-rank classification scheme based on genetic characteristics. This taxonomic framework better reflects evolutionary relationships among phages while accommodating the vast diversity discovered through metagenomic studies. For historical context and compatibility with earlier research, this review references both classical morphological classifications when discussing work conducted before these taxonomic changes and the new genetic-based system for recent studies.

## MAJOR BODY NICHES INHABITED BY PHAGES IN HUMANS

### Gut

#### General gut phageome composition and distribution

The gut represents the most extensively studied and phage-rich ecological niche in the human body, with the lower gastrointestinal tract harboring the highest viral density. Quantitative studies have shown that 1 g of feces contains 10^9^–10^10^ viral particles, reflecting the gut’s role as a major viral reservoir (8). The composition and dynamics of the gut phageome have been subject to evolving understanding. While early studies by Reyes et al. and Minot et al. suggested a predominance of lysogenic phages (9, 10), recent findings from Shkoporov et al. (11) and Bhardwaj et al. (12) reveal a more complex picture (11, 12). These latest studies emphasize that the human gut virome is highly diverse and individual-specific, with both lysogenic and lytic phages playing significant roles in shaping microbial communities.

The prevalence of lysogenic phages is evidenced by the observation that most gut bacteria harbor at least one prophage, which can be induced under various environmental conditions (13). This phenomenon has been particularly well documented in key commensal bacteria. For instance, prophage induction and secretion of myovirus or siphovirus morphotype phage particles have been observed in *Faecalibacterium* and *Bifidobacterium* (14, 15), two genera that dominate the gut microbiome at different life stages*—Bifidobacterium* being prevalent in infants and *Faecalibacterium* being more common in adults (16).

**Fig 1:**
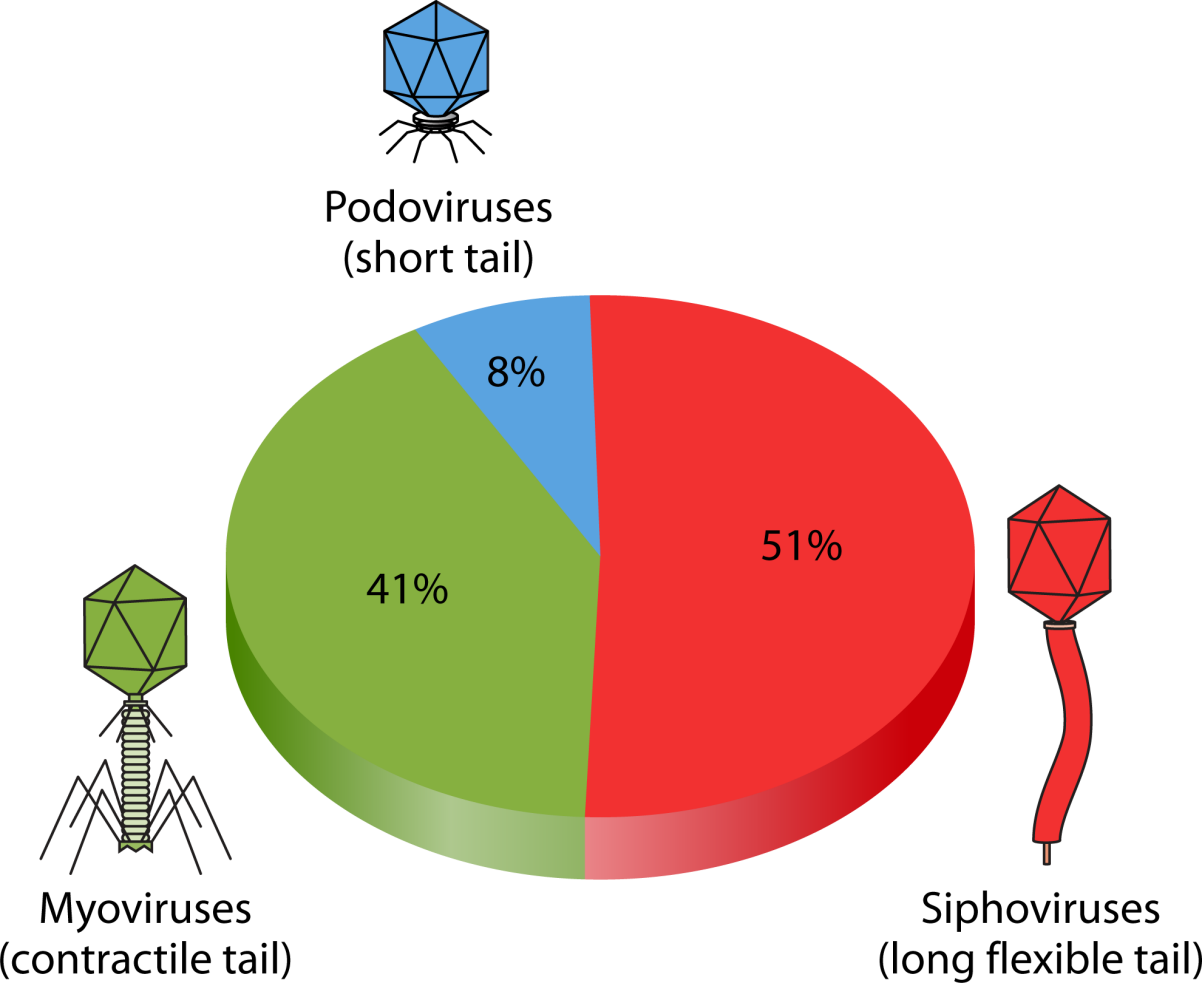
Distribution of major phage morphotypes in the human gut: siphoviruses (51%), myoviruses (41%), and podoviruses (8%). These represent the predominant viral types in healthy gut samples.

Lytic phages present in the gut are mainly represented by tailed phages with icosahedral capsids (previously: *Caudovirales*). Almost 95% of classified viral particles belong to three known morphology types: siphovirus (long flexible non-contractile tails) 51%, myovirus (long inflexible contractile tails) 41%, and podovirus (short tails) 8% (Fig. 1)(17). The phage host range mainly includes *Actinobacteria*, *Bacteroidetes*, *Firmicutes*, and *Proteobacteria* (18, 19). The human gut microbiome is characterized by a predominance of *Bacteroidetes* (primarily *Bacteroides*) and *Firmicutes* (including *Faecalibacterium*, *Eubacterium*, *Ruminococcus*, and *Clostridium*) (20, 21). Other genera such as *Bifidobacterium* contribute to this community, while *Lactobacillus* is present in lower abundances (22).

#### Gut phageome databases and resources

Recent database development has expanded our understanding of gut viral diversity. The Gut Virome Database (GVD), analyzing 2,697 viral particle and microbial metagenomes from 1,986 individuals across 16 countries, has identified 33,242 unique viral populations (23). The GVD improves virus detection compared to viral RefSeq and individual virome databases.

The Metagenomic Gut Virus (MGV) catalog, analyzed from 11,810 human gut metagenomes, has identified 189,680 viral genomes representing 54,118 candidate viral species (24). The MGV catalog improves viral detection in stool metagenomes 182-fold compared to viral RefSeq databases and increases the number of ICTV-recognized phage genera 3.5-fold. Additionally, Camarillo-Guerrero et al. (25) developed the Gut Phage Database, containing 142,000 non-redundant viral genomes (>10 kb) (25).

Shkoporov et al. (11) conducted a year-long metagenomic analysis of fecal viruses in healthy adults (11). Their study revealed that most phages were tailed phages infecting *Bacteroides*, *Faecalibacterium*, *Eubacterium*, *Prevotella*, and *Parabacteroides*. The authors classified the virome into two categories: “persistent personal virome” (PPV) and “transiently detected virome” (TDV). PPV includes bacteriophages present in the gut over months or years, while TDV consists of highly variable viral sequences of low abundance. The TDV contains phages active against *Streptococcus*, *Clostridium*, *Akkermansia*, *Acinetobacter*, *Listeria*, *Escherichia*, and *Bilophila*. In their samples, the percentage of contigs containing integrase or site-specific recombinase genes varied from 0% to approximately 70%, with most temperate phages showing siphovirus morphotype (11). Relative abundance of temperate phages, measured by contigs containing integrase and/or site-specific recombinase genes, varied from 0% to 68%.

Manrique et al. analyzed DNA sequences and metagenomic data sets from 64 healthy human gut samples (13). In over half of the samples, they identified 23 shared bacteriophages: 4 phages were classified as *Caudovirales*, 2 as *Microviridae*, 4 unclassified, and 13 incomplete genomes. They divided the healthy human gut phage community into three classes: (i) core phages common for one-half of individuals, (ii) common phages shared among 20%–50% of humans, and (iii) phages rarely shared or unique to a person (13).

#### Disease-associated gut phageome alterations

##### Colorectal cancer

Phageome composition can be altered in disorder compared to the “healthy phageome.” In the case of gut diseases, one of the most important is colorectal cancer. Hannigan et al. compared the microbiome of healthy individuals with the microbiome of adenoma and carcinoma patients (26). Colorectal cancer progresses in a stepwise process that begins when healthy tissue develops into a precancerous polyp (adenoma) that can progress to cancer if untreated (27). Using shotgun sequencing of stool samples, they found that 78.8% of viral particles were known viruses, with 93.8% being bacteriophages (mostly temperate) from *Siphoviridae* and *Myoviridae* families (26). The study identified distinct viral patterns in cancer patients compared to healthy individuals through machine learning, suggesting bacteriophages’ potential role as diagnostic biomarkers and their indirect influence on cancer development through bacterial community alteration (26, 27).

##### Liver cirrhosis

Naseri et al. studied the role of the phageome in advanced liver cirrhosis (28). The metagenomic analysis on data from data sets of fecal samples from four groups of cirrhotic patients with different etiology—(i) Chinese alcohol-induced cirrhosis group, (ii) Chinese hepatitis B virus (HBV)-related cirrhosis group, (iii) Russian patients with alcohol-related cirrhosis group, and (iv) non-alcoholic fatty liver disease group—was conducted to determine the bacteriophage composition and their functionality (28). The most prevalent phage was crAssphage (NC_024711.1) in all studied groups. About 35% phage sequences belonged to unclassified phages. The relative frequency analysis at the morphology level revealed *Siphoviridae* as the most prevalent in all cirrhotic cohorts, followed by *Podoviridae* and *Myoviridae*. In Chinese alcohol-induced cirrhosis (*N* = 10), the analyses indicated that the *Retroviridae* family was not observed, while *Enterobacteriaceae* phages, particularly targeting *Klebsiella*, were predominant. The non-alcoholic fatty liver disease group (*N* = 14) showed higher *Inoviridae* prevalence and unique presence of *Pseudomonas* virus Pf1.

Russian alcohol-related cirrhosis patients (*N* = 27) showed distinct *Leuconostoc* and *Lactococcus* siphoviruses. The Chinese HBV group (*N* = 30) had higher frequencies of specific siphoviruses: *Enterobacteria* phage cdtI, *Enterobacteria* phage mEp460, *Escherichia* virus Lambda, and *Escherichia* phage HK629 (28). The study identified 21 phage species as potential cirrhosis markers, though their presence may be affected by gut microbiota temporal dynamics (28).

##### Inflammatory bowel diseases

Other diseases linked with the gut phageome alterations are ulcerative colitis (UC) and Crohn’s disease (CD). Both of them are the long-lasting autoinflammatory diseases recognized within inflammatory bowel diseases (IBDs), with recurrent inflammation around the gastrointestinal tract (29). Genetic and environmental factors, but also intestinal microbiome, are believed to trigger the development of IBDs. (30) found that CD patients showed higher abundance of phage-hosting bacteria from *Bacteroidetes*, *Bacillales*, *Desulfovibrionales*, *Fusobacteriales*, and *Pseudomonadales* orders compared to healthy individuals (30). Additionally, phages associated with *Enterococcus*, *Ruminococcus*, *Streptococcus*, *Veilonella*, and *Parasutterella* were detected in IBD patients (31–34).

Recent studies have provided significant insights into the complex interplay between the mucosal virome and bacteriome in the pathogenesis of CD (35). In studies conducted by Cao et al., the relationship between viral and bacterial gut and chronic inflammation characteristic of CD was shown (35). The mucosal virome, both populations of lytic and temperate bacteriophages, plays a pivotal role in the progression of CD. The study revealed a significant disruption in the mutualistic interactions between bacteriophages and bacterial populations, notably those involving *Bifidobacterium* and *Lachnospiraceae*. This dysbiosis was more pronounced in CD patients during remission than during flare-ups, indicating that the imbalance persists even when clinical symptoms temporarily subside. This persistent dysbiosis underscored the refractory and recurrent nature of mucosal inflammation in CD, making it a critical area for therapeutic intervention. The findings suggest that restoring the mucosal virome could be a promising therapeutic avenue for CD. By rebalancing the virome-bacteriome ecology, it may be possible to reduce intestinal inflammation and improve disease outcomes. This approach focuses on the virome’s capacity to modulate bacterial communities and mucosal immunity, potentially offering a novel strategy to address the chronic and recurrent aspects of CD.

Given these findings about virome dysbiosis in gastrointestinal disorders, new therapeutic approaches are being explored. Gholamzad et al. highlight fecal virome transplantation (FVT) as a promising treatment for complex gastrointestinal disorders. By utilizing the microbiome’s diverse viral community, FVT has shown effectiveness in restoring gut ecosystem balance and holds potential for managing conditions such as inflammatory bowel disease and irritable bowel syndrome. This innovative approach also offers new pathways for understanding gut–virus–microbiome relationships and disease mechanisms (36).

Zuo et al. (37) found decreased tailed phage diversity in UC patients’ rectal mucosa, correlating with intestinal inflammation (37). Their analysis showed enrichment of *Escherichia* and *Enterobacteria* phages in UC mucosa compared to controls (37). Clooney et al. (2015) analyzed Norman et al.’s data sets (38, 39), revealing a “healthy” core virome in control samples that was absent in IBD patients, with temperate bacteriophages replacing the virulent core in Crohn’s disease. Environmental stress in gut diseases can trigger prophage release through reactive oxygen species activation (40).

##### Type 2 diabetes (T2D)

Nowadays, T2D is one of the most common health issues connected with human lifestyle, with too low physical activity, obesity, and high intake of highly processed food. In the long run, all that leads to insulin resistance and impaired insulin secretion and, as a result, to hyperglycemia (41). Some modifications around the bacterial part of the microbiome can lead to changes within human metabolism and cause dysfunctions connected with metabolism imbalance. Main issues linked to higher risk of type 2 diabetes are increased insulin resistance caused by reduced butyrate production in the microbiome (42) or low vitamin D production (43). Vitamin D intake is negatively linked with abundance of *Prevotella* and strongly positively associated with *Bacteroides*, both belonging to the phylum *Bacteroidetes* (44). Bacteriophages, by infecting bacteria, can potentially be one of the factors that can lead to microbiome imbalance, which reflects in metabolism changes and is connected with these illnesses (45). However, alteration in phages can also serve as an indicator for the disease state.

Chen Q et al. analyzed data from virus-like particle (VLP) sequencing of 46 samples (T2D patients and nondiabetic controls) and observed that *Myoviridae, Podoviridae, Microviridae, Siphoviridae*, and *Tectiviridae* were the most numerous phage families in fecal samples of subjects (46), with no significant differences in abundance between groups. However, *Klebsiella* and *Shigella* phages showed increased presence in the diabetic group (46). T2D correlates with higher gram-negative bacteria producing endotoxins and fewer short-chain fatty acid (SCFA)-producing bacteria (47). These endotoxins can trigger systemic inflammation affecting insulin sensitivity (47). Bacterial translocation may contribute to chronic inflammation and T2D development (48). To assess the impact of phage alterations on T2D, microbiome-related studies should be closely integrated with clinical studies.

##### Metabolic syndrome

Metabolic syndrome represents a cluster of physiological conditions that includes central obesity, insulin resistance, hypertension, elevated triglycerides, and reduced high-density lipoprotein cholesterol levels. Affecting approximately 25% of the global adult population, this metabolic disorder significantly increases the risk of developing type 2 diabetes, cardiovascular diseases, and non-alcoholic fatty liver disease. Recent evidence suggests that alterations in the gut microbiome play a crucial role in the pathogenesis of this disease, though the specific mechanisms remain under investigation.

Recent comprehensive studies of the gut phageome in metabolic syndrome patients have revealed significant alterations in the structure of the viral community (49). Analysis of 196 participants showed that metabolic syndrome is associated with decreased gut virome richness and diversity, with significant changes in specific phage families (50). Analysis of 196 participants revealed enrichment of phages infecting *Streptococcaceae* and *Bacteroidaceae*, while *Bifidobacteriaceae*-targeting phages were depleted (50). The study found that 97.4% of viral clusters were individual-specific or present in fewer than 10% of participants, with only 0.3% forming the core gut virome (50). These phage population changes correlate with alterations in their bacterial hosts, suggesting coordinated phage–bacteria dynamics in metabolic syndrome (50). These findings emphasize the personalized nature of the gut phageome, as shown by disease-specific signatures despite substantial individual variation (49). Specifically, the depletion of *Bifidobacteriaceae* phages and increase in *Bacteroidaceae* phages mirror similar changes in their bacterial host populations in metabolic syndrome patients (50).

##### Gut–brain axis and phageome

Beyond metabolic effects, recent findings indicate an intriguing connection between the gut phageome and brain function, expanding our understanding of the gut–brain axis (51). While traditionally phages were thought to influence human health primarily through their interactions with bacteria in the gut, emerging evidence suggests their impact extends to higher cognitive functions.

Recent evidence has unveiled an unexpected role of bacteriophages in cognitive function, suggesting that the influence of these viral entities extends beyond local microbiome regulation to systemic effects on host physiology, particularly brain function. Studies across multiple human cohorts have demonstrated that increased levels of specific *Caudovirales*, particularly from the *Siphoviridae* family, are associated with improved executive function and memory performance. Conversely, higher levels of *Microviridae* have been linked to cognitive impairment (51). These associations have been validated through experimental studies. Fecal microbiota transplantation from human donors with elevated *Caudovirales* levels led to improved memory performance in mice, accompanied by upregulation of memory-promoting immediate early genes in the prefrontal cortex. Furthermore, direct supplementation with *Lactococcal* 936 bacteriophages (*Siphoviridae* family) in *Drosophila* resulted in enhanced memory retention and altered expression of memory-related genes (51). The mechanisms underlying these effects appear to operate through both indirect and direct pathways: (i) indirect mechanisms involve modulation of bacterial communities and their metabolic functions, particularly folate-mediated one-carbon metabolism, and (ii) direct mechanisms suggest that bacteriophages may influence host physiology beyond their traditional role in bacterial population control (52, 53). These findings open new avenues for understanding the microbiome–brain axis and suggest potential therapeutic applications of bacteriophages in cognitive enhancement or treatment of cognitive disorders.

### Oral cavity

**Fig 2:**
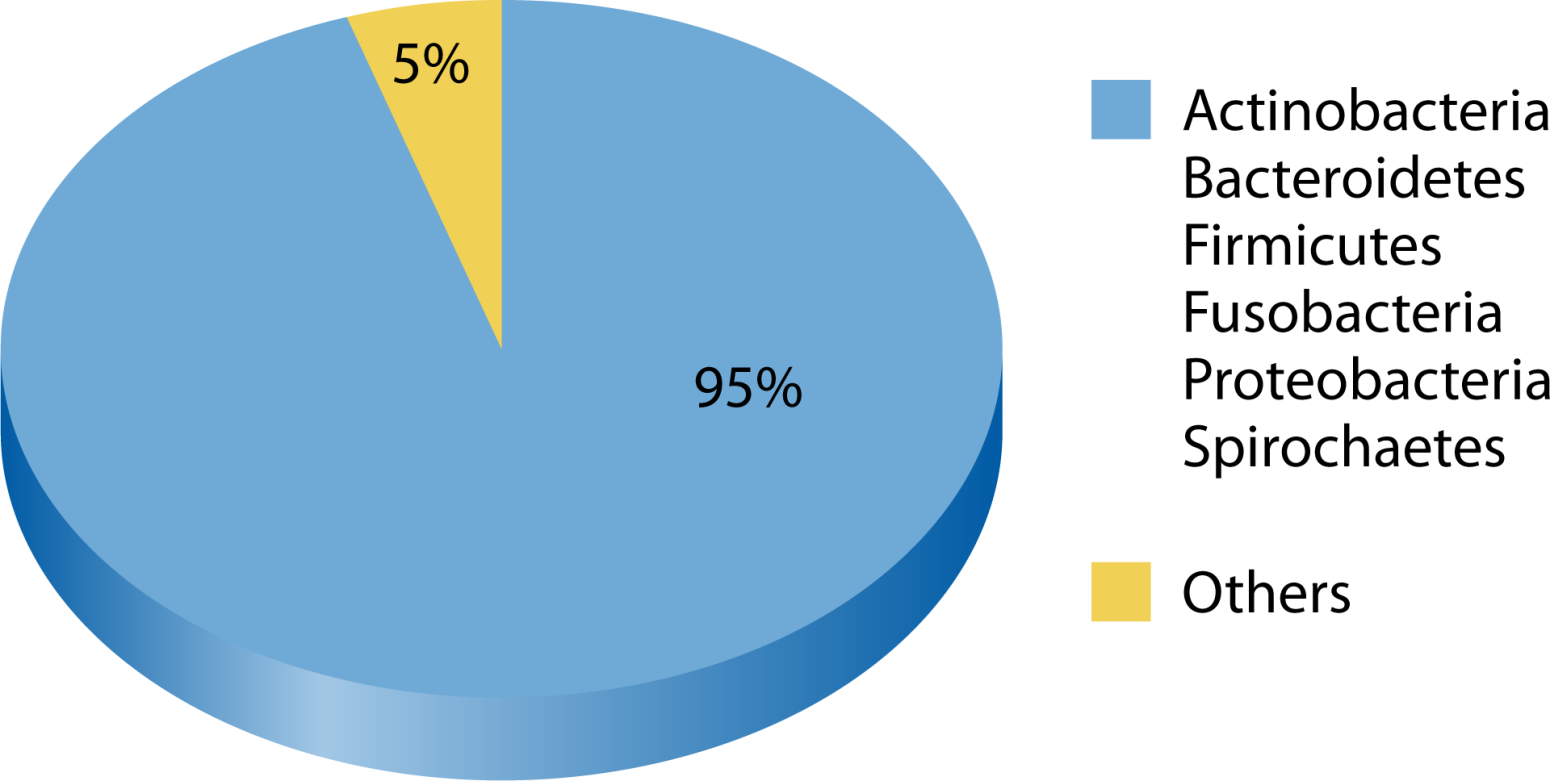
The six predominant bacterial phyla comprising over 95% of the oral microbiome, each serving as potential hosts for specific bacteriophages.

The oral cavity contains numerous microbial species in biofilms and cell clusters across periodontal tissues, tongue, and lips. One microliter of saliva contains 10^5^ VLPs, predominantly phages (54). These phages target six bacterial phyla comprising over 95% of the oral microbiome: *Actinobacteria*, *Bacteroidetes*, *Firmicutes*, *Fusobacteria*, *Proteobacteria*, and *Spirochaetes* (Fig. 2) (55). The oral cavity also contains phages of environmentally derived bacteria like *Escherichia coli*, *Pseudomonas aeruginosa*, *Staphylococcus aureus*, and *Enterococcus faecalis* (56, 57). Phages are most abundant in saliva, tooth enamel, and buccal mucosa.

Wang et al. analyzed 40 salivary and dental plaque specimens, finding *Yersinia* phage phiR1-37 (13.9%), *Lactococcus* phage KSY1 (8.4%), and *Pseudomonas* phage phiKZ (7.8%) as most abundant in saliva (58). In tooth plaque, they detected *Actinomyces* phage AV-1 (11.1%), *Streptococcus* phage (1.9%), and *Lactococcus* phage (1.6%) (58). Additional studies identified phages from *Caudovirales*, *Microviridae,* and *Inoviridae* families (59, 60).

Phages found in the oral cavity are postulated to remain stable. In Abeles et al. studies, saliva samples were studied over a 60-day period (61). The majority of the contigs had no known viral homologs. Some reads showed synteny with siphoviruses *Rhodococcus* phage Pepy6 and Poco6.

**Fig 3:**
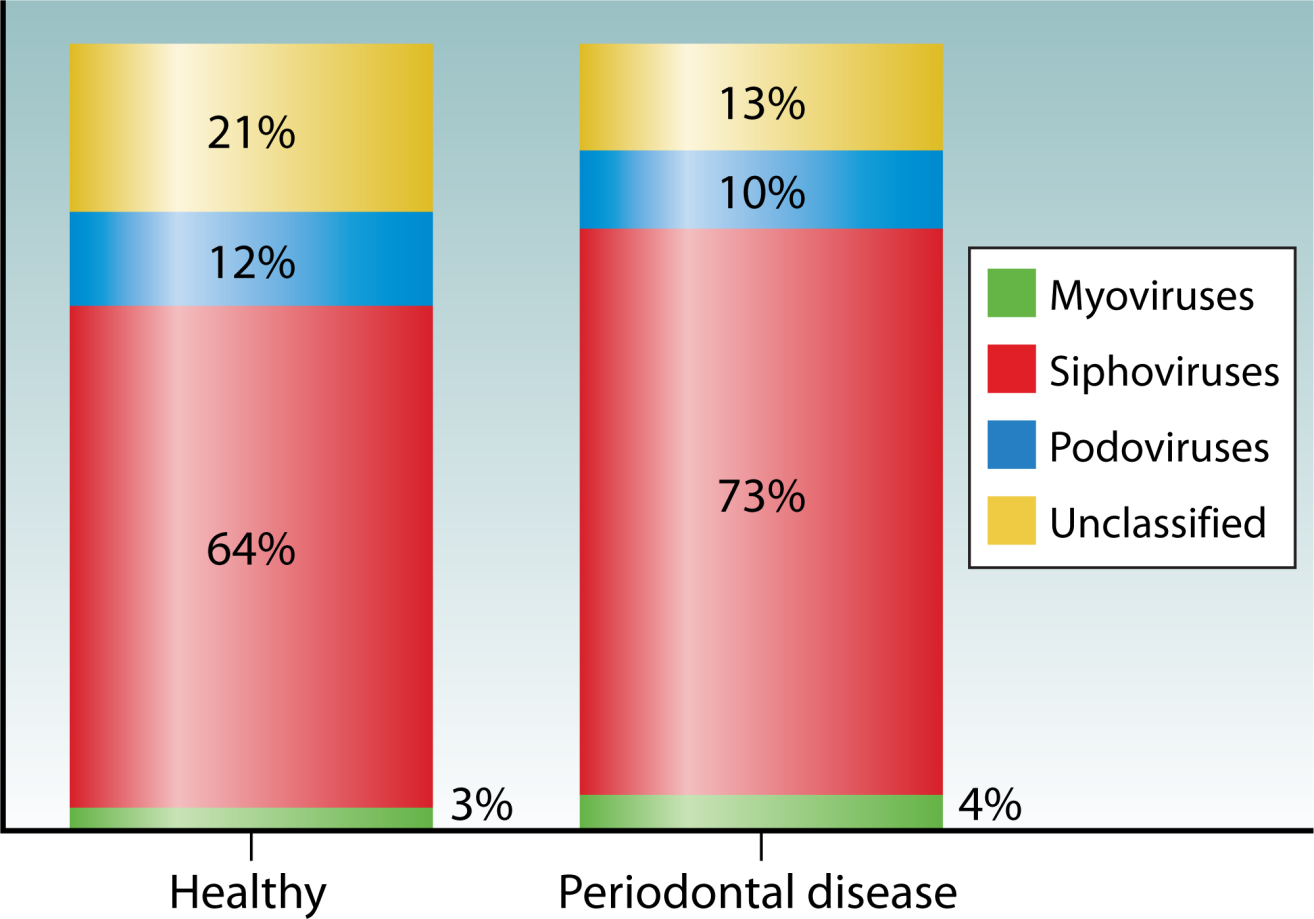
Distribution of major phage families in healthy individuals versus periodontal disease patients, showing significant differences in *Firmicutes*-targeting phages (0.5% in healthy vs 3.0% in disease, *P* < 0.001).

Major health disorders within the oral cavity are associated with the occurrence of specific pathogenic bacteria. The most common oral cavity diseases remain tooth decay (dental caries) and gingivitis, caused mainly by *Streptococcus mutans, Treponema denticola,* and *Rothia dentocariosa* (41, 62, 63). Both diseases are caused by pathogenic bacteria. Santiago-Rodriguez analyzed phage communities in 16 saliva samples (seven with periodontal disease, nine controls) (64). In healthy subjects, they found siphoviruses (64%), podoviruses (12%), and myoviruses (3%). Periodontal disease patients showed similar distributions with siphoviruses (73%), podoviruses (10%), and myoviruses (4%) (Fig. 3). Non-*Caudovirales* phages represented 7.4% in healthy and 10.6% in diseased samples. While overall phage family distributions showed no significant differences, phages targeting *Firmicutes* were significantly more abundant in disease (3.0%) compared to health (0.5%, *P* < 0.001) (64).

Willner et al. conducted a metagenomic analysis of DNA viruses from 19 pooled oropharyngeal swabs to describe the composition of oropharyngeal phage communities in healthy individuals (65). Phages were mainly observed among the population of viruses. Complete genomes were obtained for *Escherichia coli* phage T3, *Streptococcus mitis* phage SM, and *Propionibacterium acnes* phage PA6. Interestingly, metagenomic analysis revealed the presence of genes: pbIA and pbIB mediate the attachment of *Streptococcus mitis* to platelets. This phenomenon plays a significant role in *S. mitis* virulence in the endocardium. This was the first time these genes had been detected in the oral cavity (65).

### Skin

Skin contains diverse regions (moist, sebaceous, exposed, hidden) inhabited by various bacteria. Some of the most numerous in this region are *Staphylococcus* spp. and *Cutibacterium acnes* (formerly *Propionibacterium acnes*), which are predominant commensal components of the human skin microbiome (66). Microorganisms such as *C. acnes* are beneficial and serve as a physical barrier to prevent pathogen invasion. In some circumstances (broken barrier or imbalance between commensals and pathogens), a skin disease state may be developed. In case of dysbiosis of other microbiome components (e.g. commensal and pathogenic bacteria, phages), *C. acnes* can be the major etiological factor in the skin disease acne vulgaris (67).

The role of *C. acnes* in the acne pathophysiology is still discussed. Other inhabitants of the skin are phages, which are tightly connected with their bacterial hosts.

Marinelli et al. (67) focused on phages active against *Cutibacterium*. Bacterial viruses were isolated from the acne skin of a group of people unified by age and geographical location. Phages active against *C. acnes* were numerous in sebaceous regions, while *Staphylococcus* and *Streptococcus* phages were found distributed steadily on the skin surface (66). Bacteriophages of known skin inhabitants such as *Corynebacterium* phages and *Staphylococcus* phages were also observed in Hannigan’s studies (68). In the study of Hannigan et al., samples from 16 subjects at eight body sites were collected over 1 month (68). In the case of dsDNA viruses, over 90% of putative viral contigs were not taxonomically classified. Out of the remaining, most belonged to the tailed phages and were active against S*taphylococcus* spp.*, Corynebacterium* spp., and *Streptococcus* spp*.* (68, 69). They also detected environmental phages active against *Bacillus* and *Pseudomonas* (68). *Microviridae* and *Siphoviridae* were present in all skin samples, while *Podoviridae* and *Myoviridae* were less common (70).

In the case of the human skin phageome, the best studied and described are phages active against *Cutibacterium acnes*. Moreover, some studies focused on other groups of phages and their hosts indicate the presence of staphylococcal, streptococcal, corynebacterial, *Pseudomonas,* and *Bacillus* phages in this niche.

### Respiratory tract

To date, only a few studies of the respiratory tract phage communities in healthy humans have been conducted. The lower respiratory tract contains few bacteria in healthy individuals, resulting in low phage concentrations, though phages comprise the majority of identified lung viruses (71, 72).

Bacterial colonization increases during acute infections (pneumonia, bronchitis, bronchiolitis) and chronic diseases (asthma, chronic obstructive pulmonary disease, pulmonary fibrosis), providing hosts for phages. Willner et al. studied phage communities in cystic fibrosis (CF), an autosomal recessive genetic illness affecting multiple systems including the respiratory tract (73, 74). They analyzed DNA viral communities in sputum samples from CF patients and healthy individuals (71). CF is an autosomal recessive genetic illness affecting paranasal sinuses, lower respiratory, hepatobiliary, pancreatic, and lower gastrointestinal tracts (73, 74). In Wilner et al.’s studies, DNA viral communities in the samples of sputum from airways of CF patients and non-diseased individuals were analyzed (71).

Their metagenomic analysis revealed low diversity of viruses in the airways, with most viruses being uncharacterized (71). CF patients showed less variable phage communities compared to the more diverse communities in healthy individuals, possibly reflecting inhaled organisms. The host range of respiratory tract phages reflected a few dominant but distinct phages in non-diseased individuals: *Bacillus, Brucella* and *E. coli,* and *Streptococcus*. In case of patients with CF, phages active mainly against *E. coli, Streptococcus,* and *Staphylococcus* were detected (71). The increased *Staphylococcal* phages in CF patients correspond with Goerke et al.’s findings of enhanced staphylococcal prophage activation following antibiotic treatment (75). While *Pseudomonas* was present in all CF patients, their phages were not abundant in metagenomes, possibly suggesting novel phage types not detectable by current databases (75).

Further metagenomic analysis of five CF patients’ sputum showed phages mainly targeting common CF pathogens: *Burkholderia*, *Enterobacteria*, *Mycobacterium*, *Pseudomonas,* and *Streptococcus* (43). Despite high bacterial host diversity, phage populations remained similar, suggesting phages multiply on consistently present bacteria while temporary bacterial strains and their phages appear in lower amounts (43).

Bollyky’s group reported that phages active against *Pseudomonas aeruginosa* can promote the pathogenesis by this bacterium and contribute to the higher antibiotic resistance of clinical isolates of *Pseudomonas* in CF patients (76). The thick mucus coats the airways in CT patients. *Pseudomonas* forms biofilms which are resistant to antibiotic penetration. When phages active against this bacterium are present, they modify the polymer-rich biofilm into crystal structures. This drives bacterial resistance to antibiotics and masks bacteria from the elements of the immune system, thereby contributing to increased bacterial burden (77).

Interesting studies concerning the phageome in patients suffering from coronavirus disease 2019 (COVID-19) in Southern Italy were published by Ferravante et al. (78). They analyzed nasopharyngeal swab specimens from 55 patients in I (March–May 2020), II (September–November 2020), and III (January–February 2021) waves of COVID (*n* = 25, *n* = 25, *n* = 5, respectively). Analysis of taxonomic profiling data revealed that phage families belonging to the *Caudovirales* are abundant in the patient samples. *Siphoviridae* morphology phages were the most common, even nine times higher than the second most rich family *Microviridae* (78).

### Urogenital niche

**Fig 4:**
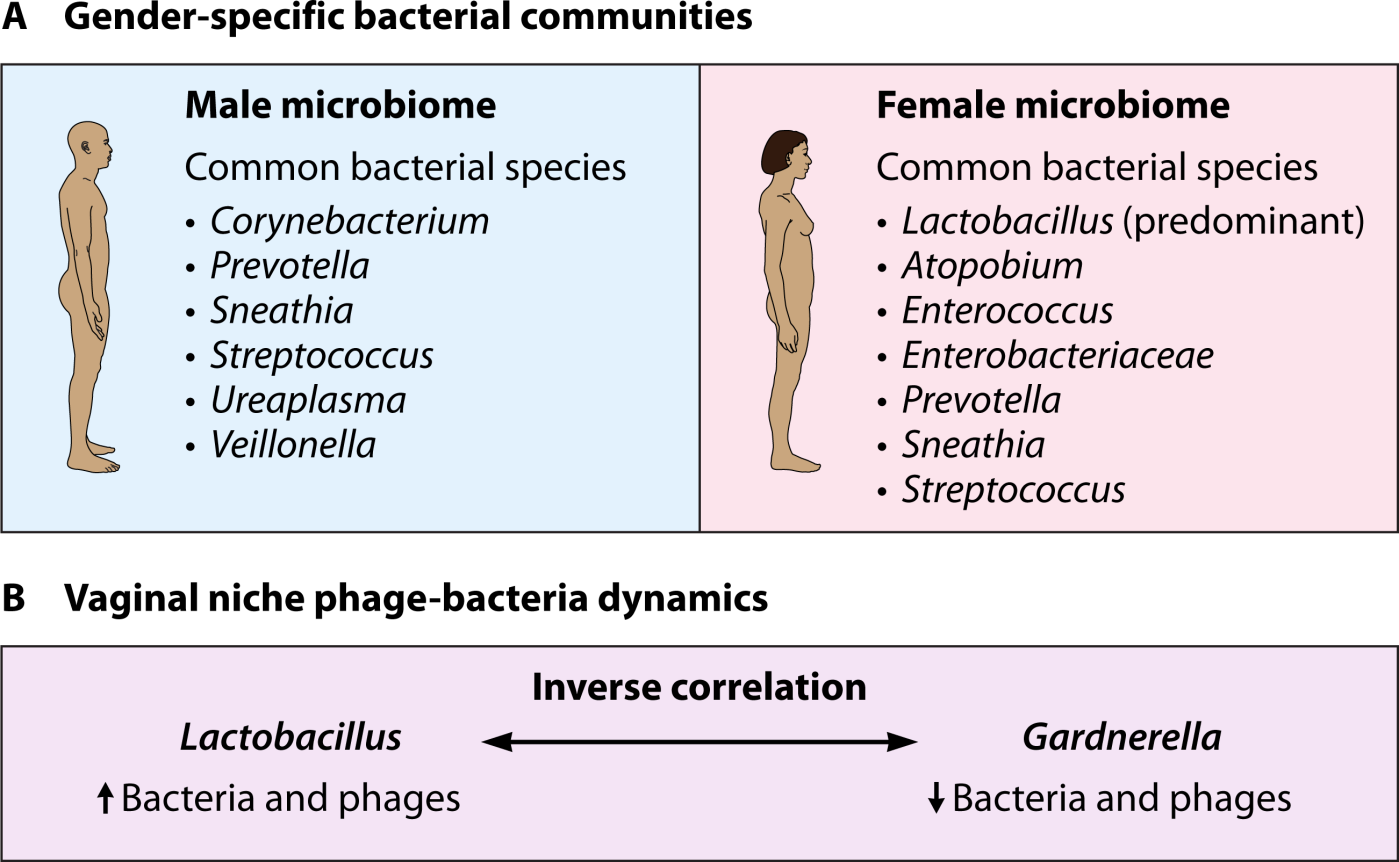
Comparison of predominant bacterial genera between male and female urogenital tracts, illustrating gender-specific microbiome composition that influences associated phage communities.

The human urogenital niche carries specific microbiomes. Furthermore, discrepancies in bacterial species and related phages were observed in women and men. Significant differences between female and male genitourinary tracts were studied: in men, the most common bacterial species are *Corynebacterium*, *Prevotella*, *Sneathia*, *Streptococcus*, *Ureaplasma*, and *Veillonella* (79, 80). In women, *Atopobium*, *Corynebacterium, Enterococcus*, *Enterobacteriaceae*, *Lactobacillus*, *Prevotella*, *Sneathia*, and *Streptococcus* dominated (81). In the case of women’s vaginal niche, *Lactobacillus* dominates other bacteria (81, 82). Therefore, many phages (both temperate and lytic) active against this bacterium were isolated from this tract (82). Interestingly, the presence of *Lactobacillus* phages is negatively correlated with phages targeting *Gardnerella vaginalis*, which is a component of the dysbiotic vaginal microbiome (83). While the number of probiotic bacteria (*Lactobacillus*) and their phages increases, the number of pathogenic bacteria (*Gardnerella*) and phages active against them decreases (Fig. 4).

The main challenge in the studies of a urinary phageome is sampling, as DNA concentrations are often low and may require amplification prior to sequencing (84). Brown-Jacque et al. found sample contamination from skin phages during collection (85). Studies comparing catheterization and suprapubic aspiration have shown that both methods successfully avoid vulvo-vaginal contaminants and yield samples distinct from skin or vaginal microbiomes. Voided urine samples, while less invasive, routinely show vaginal contamination even with clean-catch protocols. Therefore, investigations of the bladder phageome should ideally use urine obtained by catheterization or suprapubic aspirate until less invasive but equally reliable collection methods are developed (86).

Santiago-Rodriguez et al. compared viromes between 10 urinary tract infection (UTI) patients and 10 healthy controls (64). Only 27% of identified contigs matched known phage sequences, with reads mapping to phage PH15, *E. coli* phage phiV10, and *Enterococcus* phage phiFL4A (64). While no significant differences correlated with urological health, they found high prevalence of integrases, suggesting substantial lysogenic phage presence. Moustafa et al. analyzed 49 UTI samples, detecting phages targeting common urinary tract pathogens including *Enterobacteria*, *Enterococcus*, *Escherichia*, *Lactobacillus*, *Propionibacterium*, *Salmonella*, *Shigella*, *Staphylococcus*, *Streptococcus*, *Pseudomonas*, *Vibrio*, *Yersinia*, and Stx2-converting bacteriophages (87).

In summary, urogenital microbiome and phageome composition shows clear gender specificity. Therapeutic applications of phages in this niche include standalone use (39%), combination with antibiotics (18%), and unspecified approaches (43%), primarily via intravesicular administration (84). The vaginal phageome particularly influences microbiome-related conditions, serving as a potential predictor for bacterial vaginosis (88).

## CONCLUSIONS

The human body harbors diverse microbial communities including bacteriophages, with composition varying by age, sex, geographical region, and individual characteristics. Bacteriophages significantly influence human health by maintaining microbiome stability, particularly in the oral cavity and gastrointestinal tract, while showing more transient presence in the urinary tract, skin, and lungs (Table 1).

**TABLE 1:** Major phageome characteristics across body sites

Body site	Predominant phage types	Key host bacteria	Typical viral density	Notable features
Gut	*Siphoviridae* (51%) *Myoviridae* (41%) *Podoviridae* (8%)	*Bacteroides*, *Faecalibacterium*	10^9^–10^10^ VLP/g	Highest viral density
Oral cavity	*Siphoviridae* predominant	*Streptococcus*, *Actinobacteria*	10^5^ VLP/μL	Stable communities
Skin	Varied by region	*Cutibacterium*, *Staphylococcus*	Variable	Site-specific distribution
Respiratory	Limited diversity	*Pseudomonas*, *Streptococcus*	Low in healthy state	Disease-dependent
Urogenital	Gender-specific	*Lactobacillus, Streptococcus*	Variable	Sexual dimorphism

Recent studies demonstrate that phage populations can serve as indicators of pathological states and potential disease predictors for conditions including colorectal cancer, ulcerative colitis, Crohn’s disease, and type 2 diabetes. While these correlations are well-documented, the underlying mechanisms remain unclear. The phageome’s influence appears to operate through both direct effects on human physiology and indirect modulation of bacterial communities, raising fundamental questions about causality in disease processes.

When discussing the literature on bacteriophage composition, host assignment, and phage lifestyle, it is essential to highlight the sample preparation methods used for virome analysis in these studies. Some studies have utilized total microbial DNA for phageome analysis, while others have employed DNA from purified VLPs. The choice of method significantly impacts the detection and characterization of different phage populations, particularly in distinguishing between active virions and integrated prophages. This methodological variation must be considered when interpreting and comparing results across studies, as each approach provides different insights into phage ecology and lifestyle.

The analysis of current literature reveals several emerging trends and critical knowledge gaps. Promising developments include the recognition of phage roles in cognitive function through the gut–brain axis and the development of therapeutic approaches like fecal virome transplantation. However, significant challenges persist: the mechanisms of phage-mediated bacterial community regulation in different body sites remain poorly understood; the factors controlling the switch between lytic and lysogenic cycles *in vivo* need clarification; and the extent of direct phage–human cell interactions requires further investigation. Additionally, standardization of sampling, sequencing, and analysis methods across studies would enable more robust conclusions about phage community dynamics.

Understanding these complex relationships could provide new approaches for disease prediction, diagnosis, and treatment. Future research should focus on establishing causal relationships between phageome alterations and disease states, developing standardized methodologies for phageome analysis, and exploring therapeutic applications that leverage our growing understanding of phage–host interactions (Table 2).

**TABLE 2:** Current research gaps and future directions

Research area	Current limitations	Future directions
Methodology	Sampling challenges, lack of standardization	Development of standardized protocols
Host range	Limited understanding of host specificity	Advanced host prediction tools
Therapeutic applications	Limited clinical trials	Controlled studies for phage therapy
Mechanistic understanding	Unclear causal relationships	Multi-omics integration studies
Temporal dynamics	Limited longitudinal studies	Long-term monitoring studies
